# Supplementary material for: The genome sequence of the plant bug, Alydus calcaratus (Linnaeus, 1758) (Hemiptera: Alydidae)
Source: Wellcome Open Res. 2025 Oct 3;10:533. [Version 1] doi: 10.12688/wellcomeopenres.24921.1 (PMC12741555; doi:10.12688/wellcomeopenres.24921.1)
Supplement: Peer review history

# Reviewer report: Reviewer response for version 1

**DOI:** 10.21956/wellcomeopenres.27451.r142905

**Published:** 2025-12-26

**Authors:** Abdoallah Sharaf

**Affiliations:** 1 University of Konstanz, Konstanz, Germany

The article is scientifically sound in its current form, with no major flaws in rationale, methodology, reproducibility, or data presentation. All evaluation criteria can be confidently answered “Yes,” reflecting high technical quality, methodological transparency, and strong data integrity throughout the study.

 The work adheres closely to the standards and best practices of the Darwin Tree of Life (DToL) and Earth BioGenome Project (EBP) initiatives, delivering a high-quality, chromosome-level reference genome for *Alydus calcaratus*. The assembly demonstrates excellent performance metrics, including near-complete chromosomal assignment (99.21% scaffolded into 7 pseudomolecules), a scaffold N50 of 152.52 Mb, BUSCO completeness of 98.4% (Hemiptera odb10), and high consensus quality values (QV ≈ 58). These results indicate careful experimental design, robust sequencing depth, rigorous assembly curation, and thorough quality control.

 The methodological description is comprehensive and reproducible, covering specimen provenance, DNA extraction, PacBio HiFi and Hi-C sequencing, assembly, curation, and validation in sufficient detail. The integration of k-mer–based analyses, BUSCO, Merqury, Hi-C contact maps, and BlobToolKit visualisations provides a transparent and convincing assessment of assembly completeness and accuracy. Identification and reporting of a heterozygous inversion further demonstrate careful manual curation rather than uncritical pipeline output.

 No substantive revisions are required to support the scientific soundness or validity of the work. Overall, this genome sequence note represents a valuable and well-executed contribution to hemipteran genomics, biodiversity genomics, and the growing genomic resources generated by the Darwin Tree of Life Project.

Are sufficient details of methods and materials provided to allow replication by others?

Yes

Is the rationale for creating the dataset(s) clearly described?

Yes

Are the datasets clearly presented in a useable and accessible format?

Yes

Are the protocols appropriate and is the work technically sound?

Yes

Reviewer Expertise:

genomics, bioinformatics, and biodiversity research

I confirm that I have read this submission and believe that I have an appropriate level of expertise to confirm that it is of an acceptable scientific standard.

# Reviewer report: Reviewer response for version 1

**DOI:** 10.21956/wellcomeopenres.27451.r137266

**Published:** 2025-11-21

**Authors:** Roberto Biello

**Affiliations:** 1 John Innes Centre Department of Crop Genetics, Norwich, England, UK

The authors present a high-quality, chromosome-level genome assembly for *Alydus calcaratus*, generated using PacBio HiFi and Hi-C sequencing technologies. This assembly, which includes both haplotypes, represents a valuable genomic resource for studying the evolution of species adapted to unique ecological niches such as coastal areas and sand dunes. The assembly meets high-quality standards, as demonstrated by high BUSCO scores and k-mer completeness. The methods are clearly described, and all associated data are publicly available.

 Minor comments:

 1. The manuscript reports the presence of 6 autosomes and an X sex chromosome system. Is this karyotype consistent with previously published cytogenetic data for this species or related species? Furthermore, chromosome 6 is notably small (~10 Mb) and has a higher GC content. Could the authors comment on whether this was an expected finding and its potential biological significance, if any?

 2. In Figure 3, the scaffold name for the last chromosome on the y-axis appears to be missing. Please ensure all labels are present for clarity.

Are sufficient details of methods and materials provided to allow replication by others?

Yes

Is the rationale for creating the dataset(s) clearly described?

Yes

Are the datasets clearly presented in a useable and accessible format?

Yes

Are the protocols appropriate and is the work technically sound?

Yes

Reviewer Expertise:

Evolutionary biology, conservation genomics

I confirm that I have read this submission and believe that I have an appropriate level of expertise to confirm that it is of an acceptable scientific standard.
